# Vulnerable families and costly formula: a qualitative exploration of infant formula purchasing among peri-urban Peruvian households

**DOI:** 10.1186/s13006-021-00356-6

**Published:** 2021-01-19

**Authors:** Jessica D. Rothstein, Peter J. Winch, Jessica Pachas, Lilia Z. Cabrera, Mayra Ochoa, Robert H. Gilman, Laura E. Caulfield

**Affiliations:** 1 Department of International Health, Johns Hopkins Bloomberg School of Public Health, Baltimore, MD USA; 2 Asociación Benéfica Proyectos en Informática, Salud, Medicina, y Agricultura (PRISMA), Lima, Peru; 3 Universidad Peruana Cayetano Heredia, Laboratorio de Investigación en Enfermedades Infecciosas, Lima, Peru

**Keywords:** Infant feeding, Breast milk substitutes, Decision-making, Qualitative, Peru

## Abstract

**Background:**

Substantial evidence exists surrounding the health risks of breast milk substitutes (BMS) in place of exclusive breastfeeding among infants < 6 months of age in resource-poor settings. Yet, mothers’ experiences of selecting and purchasing BMS brands have not been well studied to date. This qualitative study explored the factors influencing BMS purchasing practices, along with the consequences of those decisions, in peri-urban Lima, Peru.

**Methods:**

We conducted in-depth interviews (IDIs) with 29 mothers who had begun mixed-feeding their infants during the first 6 months of life. Interviews explored participants’ reasons for initiating infant formula use and their experiences of selecting, purchasing, and providing BMS to their children. Audio recordings were transcribed, coded, and key themes and illustrative vignettes were identified.

**Results:**

The primary reported reasons for initiating infant formula use included having received a recommendation for infant formula from a healthcare provider, concerns about an infant’s weight gain, and the perception of insufficient breast milk. Mothers tended to initially purchase the BMS brand that had been recommended by a doctor, which was often more expensive than the alternatives. The costs of BMS, which escalated as infants grew, often disrupted the household economy and generated significant stress. While some mothers identified alternatives allowing them to continue purchasing the same brand, others chose to switch to less expensive products. Several mothers began to feed their infants follow-on formula or commercial milk, despite their awareness that such practices were not recommended for infants under 6 months of age. The approval of family members and the absence of an infant’s immediate adverse reaction influenced mothers’ decisions to continue purchasing these products.

**Conclusions:**

The high costs of BMS may deepen existing socio-economic vulnerabilities and generate new risks for infant health. The continued dedication of resources towards breastfeeding education and support is critical, and strategies would benefit from underscoring the long-term financial and health consequences of infant formula use, and from strengthening women’s self-efficacy to refuse to initiate infant formula when recommended. In addition, health providers should be trained in counseling to help women to relactate or return to exclusive breastfeeding after cessation.

## Background

The global promotion of breast milk substitutes (BMS) continues to threaten optimal breastfeeding practices, with consequences for the health of infants, mothers, and societies at large [1–3]. Worldwide, less than 40% of children are breastfed exclusively through 6 months of age, as recommended by the World Health Organization (WHO) [4, 5]. Compared to infants < 6 months that consume only breast milk, infants consuming BMS either exclusively or in combination with breast milk (i.e., mixed feeding) face higher risks of infectious morbidity and mortality during the first 2 years of life and are more susceptible to chronic diseases over the long term [6–10]. Reductions in the exclusivity and duration of breastfeeding, which may lead to or result from BMS use, have implications for maternal health as well, given that breastfeeding is associated with protections against ovarian cancer, breast cancer, and type 2 diabetes [11, 12]. At the societal level, reduced breastfeeding prevalence is associated with lower intelligence scores, resulting in poorer educational attainment and earning potential in adulthood [13, 14]. It is estimated that the economic cost of lower cognition resulting from suboptimal breastfeeding amounts to $70.9 billion in low- and middle-income countries (LMIC) [15].

Conventional wisdom held that poverty would “protect” breastfeeding, based on the assumption that poor families could not afford BMS [16]. However, recent research indicates that the rising prevalence of infant formula feeding is particularly pronounced among poor populations, partially in response to the infant formula industry’s aggressive marketing strategies in “emerging economies” [17, 18]. Evidence is also mounting that these companies specifically target the most economically vulnerable mothers through direct-to-consumer advertising and indirectly through the health system [17, 19–21].

A number of studies have examined how the financial burden of BMS use leads to practices that may create additional health risks for infants. In South Africa and Indonesia, analyses of the protein concentrations of infant formula feeds revealed that approximately one-third of samples were severely over-diluted, making them nutritionally inadequate [22, 23]. Among low-income populations in the U.S., mothers have engaged in “formula stretching” by adding cereals to the bottles or by increasing the time intervals between feeds [24–27]. Also, concerns about wasting expensive infant formula in resource-poor settings lead to prolonged periods of storage of prepared feeds at room temperature, which supports rapid bacterial growth [28–30].

In addition to affecting how infant formula is prepared and handled within the home, socioeconomic constraints may also influence caregivers’ selection and purchasing of BMS brands. Understanding these decision-making processes is critical to characterizing the full implications of BMS use for both infants and families. Nevertheless, infant formula brand selection and purchasing behaviors of women in resource-poor settings have not been well examined to date.

### Study background

In Peru, prevalence of exclusive breastfeeding (EBF) among infants under 6 months of age exhibited large increases during the 1980s and 1990s, following the adoption of the International Code of Marketing of Breast-milk Substitutes in 1982 along with increased investment in training health workers to support breastfeeding [31–33]. Breastfeeding initiation remains a social norm in Peru, with an estimated 93.0% of infants breastfeeding within the first 24 h after birth [34], but progress towards increasing breastfeeding duration has slowed since 2000. According to the most recent Demographic and Health Survey, the percentage of breastfeeding infants under 6 months of age remained steady between 2000 and 2018, with a modest decrease from 67.2 to 66.4%, respectively [34]. Median duration of any breastfeeding has also declined, from 21.7 months in 2000 to 20.7 months in 2016 [35, 36]. Meanwhile, prevalence of infant formula use in children under 2 months of age have risen slightly from 17.2% in 2004 to 19.4% in 2016 [37, 38]. Total infant formula sales in Peru grew by 159.7% in Peru between 2008 and 2013, as compared to an average rate of growth of 40.8% in all LMIC [1].

Previously, our research team analyzed daily surveillance records from a newborn cohort study in peri-urban Lima, Peru in order to characterize patterns of mixed feeding during the first 2 months of life. Among a sub-set of 214 mother-infant dyads, we found that that nearly half (47.2%) of all infants received mixed feeding within the first 60 days of life, after which breastfeeding prevalence roughly stabilized.

Building on these findings, we conducted cross-sectional research consisting of semi-structured questionnaires and in-depth interviews (IDIs) to examine the social determinants of mixed feeding among study participants. Of 214 mothers, 73 (34.1%) had received a verbal recommendation for infant formula from a health provider, and most of these mothers also received a written prescription for a specific brand. Infant formula recommendations were strongly associated with infant formula use: mothers who received an infant formula recommendation were over ten times more likely to practice mixed feeding during the first 60 days of the infant’s life, even after adjusting for other significant predictor variables. Qualitative data collected from IDIs with mothers and health providers revealed that infant formula industry representatives often entered local health facilities and engaged with providers, in violation of the International Code of Marketing of Breast-milk Substitutes [31, 39]. According to both doctors and nurses, these representatives would leave infant formula samples for distribution to patients, promotional items such as calendars to be displayed in clinic rooms, and would offer gifts, such as free or subsidized conferences, to incentivize providers to promote their products. Meanwhile, breastfeeding promotion efforts (largely performed by nurses) were weakened by insufficient counseling, and mothers generally viewed nurses as inattentive and less trustworthy as compared to doctors. We concluded that these two health system factors; (1) providers’ recommendations and prescriptions for infant formula, seemingly resulting from infant formula industry activities, and (2) inadequate facility-based breastfeeding promotion, gave rise to maternal attitudes that resulted in decisions to begin mixed feeding [39].

### Study objectives

The theme of costly infant formula emerged during data collection for the aforementioned cross-sectional study. This led us to conduct additional analyses of the interview data to better understand the interplay between socio-economic factors, health providers’ influence, BMS use, and BMS brand selection and purchasing practices. Here we report on findings from interviews with 29 mothers in order to explore factors influencing BMS purchasing practices, and the effects of those decisions, among low-income households in peri-urban Lima.

## Methods

### Participants and sample selection

This qualitative study was nested within a larger newborn cohort study in the shantytown district of *Villa El Salvador* on the southern outskirts of Lima, Peru. The larger cohort study took place in four of *Villa El Salvador*’s ten geographical sectors, which vary considerably in terms of access to infrastructure and household socio-economic status. Poverty and food insecurity remain widespread throughout the district [39].

Mothers participating in the cohort study with infants aged < 9 months, who had begun mixed feeding before the infant reached 6 months, were eligible to participate in the qualitative research. Infants with very low birthweight (< 1500 g) and those with a severe chronic, congenital, or neonatal diseases were excluded from the cohort study. For the purposes of the cohort study, field workers conducted daily surveillance activities that included documenting each study participant’s infant feeding status over the past 24 h. Eligible participants for the qualitative study were identified through ongoing review of these surveillance records. We aimed to recruit approximately 30 interview participants; this sample size was deemed sufficient to reach thematic saturation based on criteria outlined by Malterud et al., including relatively specific aims, the application of a theoretical framework, and strong interview dialogue [40]. Given that the study population exhibited minimal ethnic and linguistic diversity, a larger sample size was not deemed necessary. The response rate was 93.5%, as two of the 31 eligible women selected from the cohort study declined to participate in the IDI.

We sampled purposively to recruit a minimum of five participants from each geographical sector, given their socio-economic heterogeneity, and to achieve variability across maternal characteristics such as age, educational attainment, and parity. This sampling strategy was thought to ensure that a variety of perspectives, including both average and unusual cases, would be represented in our data.

To recruit participants, cohort study field workers introduced the nested qualitative study to selected participants during home visits for the cohort study’s daily surveillance activities. If a participant expressed interest in participating in the IDI, the first author and a field worker subsequently conducted a separate home visit to explain the goals, procedures, and requirements of the interview in more detail. Upon agreeing to participate, the field worker scheduled a time for the interview based on the participant’s availability. Written informed consent was obtained by a field worker on the day of the interview.

### Definitions of terms

For the purposes of this study, the term “breast milk substitute” denotes any liquid that is marketed or in any way represented as suitable to be a partial or full replacement for breast milk, in accordance with the WHO definition [31, 41]. This includes both infant formula, which specifies an age of introduction of 0–6 months, as well as follow-on (or follow-up) formula, which specifies an age of introduction of > 6 months. In this paper, the term follow-on formula includes products that are variably referred to as “growing up milk” or “toddler’s milk” and are marketed for children 12–36 months of age [42]. Other types of milk that are not explicitly or implicitly indicated for infants and young children are referred to as commercial milk (Table 1).

**Table 1:** Definition of terms for breast milk substitutes

Term	Definition	Example
Infant formula	Formula marketed as a breast milk substitute for children 0–6 months	*Enfamil*
Follow-on formula	Formula/milk with an indicated age of introduction of > 6 months; “growing up milk” or “toddler’s milk” usually marketed towards children 12–36 months	*Enfagrow*
Commercial milk	Milk (liquid or powdered) for average adult consumer	*Anchor*

### Data collection

In-depth interviews followed a semi-structured format and were steered by an interview guide that provided open-ended questions and follow-up probes. To explore how infant feeding practices and perceptions took shape over time, interview topics included childbirth, early breastfeeding efforts, participants’ decisions to initiate formula use, and the experiences of selecting, purchasing, and providing formula to their children. To understand the influences on mothers’ decision-making surrounding feeding and purchasing behaviors, we aimed to consider factors operating at several ecological levels [43]. Thus, interview questions focused not only on maternal and infant attributes, but also on the various sources of information related to breastfeeding and infant formula use, including clinical encounters, household members, and participants’ social networks. In addition, certain interview questions were informed by prospect theory, which focuses on the role of message framing in shaping people’s decision-making behaviors [44]. Thus, we examined how positive “gain” and negative “loss” framing may influence BMS purchasing practices [44, 45]. The interview guide was modified over the course of the study to probe on emerging themes, and continuous feedback from cohort study field workers ensured that questions were culturally acceptable and clear.

The first author conducted the interviews in Spanish with assistance from a field worker, who at times served as a secondary interviewer. The field worker’s presence helped participants speak candidly about their experiences, given that she was from *Villa El Salvador* and similar in age to many of them. Most women participated in a single interview, but follow-up interviews were conducted with three women in order to clarify responses from the original interviews and probe further on emerging thematic areas. Later-stage interviews were also used as respondent validation, or “member-checking,” in which preliminary analyses were presented to mothers for feedback [46]. Interviews were digitally recorded with the participant’s consent. Pseudonyms are used for quotations in presentation of results.

Sociodemographic data on study participants were collected upon enrollment into the cohort study and through supplemental questionnaires. These included maternal (age, education, civil status, parity) and infant (sex, birthweight) characteristics, and household socio-economic data. Food insecurity was assessed through the Household Food Insecurity Access Scale (HFIAS), a 9-item questionnaire adapted by USAID’s Food and Nutrition Technical Assistance project for use among low-income households in a variety of cultural contexts [47].

### Data analysis

We analyzed data on an ongoing basis throughout the data collection period. Digital recordings were transcribed verbatim, supplemented with extended field notes, and analyzed directly from Spanish so as to avoid losing the nuances of the data. An initial coding framework was developed consisting of both a priori codes and emergent codes surfacing from the text. This led to the development of a codebook including code definitions, guidelines on when to apply codes, and example quotes, which was reviewed and refined by several research team members. The first author applied final codes to the transcripts using ATLAS.ti version 7.0 (2012) qualitative data management software (Scientific Software Development, Berlin, Germany).

Following the procedures for qualitative content analysis described by Graneheim and Lundman, codes were organized into categories and sub-categories that reflected the “manifest content” or visible elements of the text [48]. The first author then linked the underlying meanings and relationships among categories into broader themes, expressing the text’s “latent content” [48]. Throughout the iterative process of data collection and analysis, the first author wrote successive analytic memos to explore emerging themes and reflect on early interpretations of the data [49]. In addition, the first author elicited feedback from the team of field workers during various stages of analysis in order to confirm sound interpretations and to fill in contextual gaps [50]. Upon completion of data collection, the first author returned to each transcript to confirm support of the themes by the codes and search for any disconfirming data.

### Ethical approval

The research protocol was approved by the institutional review board at the Johns Hopkins Bloomberg School of Public Health (Baltimore, MD), Johns Hopkins University IRB number: 00008609 and the ethics committee at Asociación Benéfica PRISMA (Lima, Peru), the local collaborating institution. Written informed consent was obtained from all study participants by the field worker directly preceding the interview; if the mother was < 18 years of age (*N* = 2), written informed consent was provided by the infant’s grandmother and oral assent was obtained from the mother.

## Results

### Participant characteristics

A total of 29 mothers participated in the in-depth interviews. Mothers were on average 27 years old, married or cohabiting, multiparous, and had 12 years of schooling (Table 2). Infants weighed an average of 3139 g at birth (SD = 785), with one classified as low birthweight (LBW; < 2500 g), and 44.8% were male. Ten households (34.5%) lacked property rights (i.e., did not have an official title to their land), and 17 (58.6%) were moderately or severely food insecure, according to the HFIAS classification.

**Table 2:** Participant characteristics

Variable	Mean +/− SD or N (%)
*Maternal socio-demographic characteristics*	
Maternal age, years	27 +/− 8.1
Maternal educational attainment	
Incomplete primary school (< 6 years)	4 (13.8)
Incomplete secondary school (6–11 years)	8 (27.6)
Complete secondary school (12 years)	17 (58.6)
Civil status (single)	5 (17.2)
Parity (primiparous)	5 (17.2)
Property ownership (without land title)	10 (34.5)
Food insecurity access	
Food secure	8 (27.6)
Mildly food insecure	4 (13.8)
Moderately food insecure	10 (34.5)
Severely food insecure	7 (24.1)
*Infant and infant feeding characteristics*	
Type of delivery	
Vaginal	17 (58.6)
Cesarean	12 (41.4)
Infant birthweight, grams	3139 +/− 785
Infant sex (male)	13 (44.8)
`Age of child upon initiation of formula use	
` < 1 week	12 (41.4)
More than 1 week, less than 1 month	10 (34.5)
1–3 months	5 (17.2)
3–6 months	2 (6.9)

All infants in our sample were initially breastfed, and the majority (75.9%) of infants were introduced to infant formula during the first month of life (Table 2). More than 40% of mother’s initiated infant formula use before their infants were 1 week old (Table 2). After beginning mixed feeding, most study participants continued to breastfeed during the first 6 months of their infant’s life; only three participants ever fed their infants exclusively with BMS for an extended period of time, starting when their infants were between 3 and 5 months of age.

### Overview of available BMS and commercial milk in peri-urban Peru

Four brands of BMS were commonly available and purchased by the study population: *Enfamil*, *NAN*, *Similac* and *S-26 GOLD*. The manufacturers and target child age ranges of different brands are displayed in Table 3. All of these products are sold as dehydrated powders to be mixed with water upon preparation. Several brands market a number of different formulations and specialty lines with large variations in price. For example, the *Enfamil* line includes the standard product (*Enfamil con Hierro* or “Enfamil with Iron”), a premium product (*Enfamil Premium*), an easily digestible version (*Enfamil Confort* or “Enfamil Comfort”), and an anti-constipation version (*Enfamil Anti-Estriñimiento*).

**Table 3:** Common brands of BMS available in study site

Brand and type^a^	Target child age range	Manufacturer
Enfamil Premium 1	0–6 months	Mead Johnson & Company (USA)
Enfamil con Hierro (with iron) 1	0–6 months	
Enfagrow	12–36 months	
NAN 1	0–6 months	Nestlé (Switzerland)
NAN 2 (liquid)	6–12 months	
Similac	0–6 months	Abbott Laboratories (USA)
S-26 GOLD	0–6 months	Aspen Nutritionals (Australia)

^a^All formula types are sold as dehydrated powder unless otherwise noted

These brands also offer follow-on formulas, such as the *Enfamil* line, *Enfagrow*. *NAN* offers follow-on formulas sold as liquid in a small tin (410 g) containing one to two days’ worth of infant formula (*NAN 2* and *NAN 3*). Two brands of commercial milk were widespread in the study communities: *Anchor*, produced by Nestlé, was sold in powdered form, while *Gloria* milk, produced by the Peruvian company Leche Gloria S.A., was sold as a liquid.

### Reasons for commencing formula use

Participants described various experiences and interactions that motivated their decisions to begin mixed feeding. Of 29 mothers, 19 (65.5%) received a verbal recommendation for infant formula from a health provider (Table 4), often without prompting. In these cases, participants reported that doctors either preemptively suggested that she consider infant formula use if she were to have trouble breastfeeding in the future, or suggested infant formula use after a participant mentioned a problem or concern. The one exception involved the mother of the only LBW baby in our sample, who reported that a doctor told her upon hospital discharge that it was medically necessary to give infant formula with breast milk to help the infant grow. Along with the recommendations, thirteen women also received written prescriptions, which included the name of a specific infant formula brand and instructions for use. Aside from one case in which the prescription was accompanied by a voucher for free infant formula after the purchase of one tin, the prescriptions did not provide price discounts.

**Table 4:** Reported reasons for initiating formula use

Variable	Total N ***N (%)***^b^	Age of child upon initiation of formula use ***N (%)***^c^			
		< 1 week (***N*** = 12)	> 1 week, < 1 mo. (***N*** = 10)	1–3 mo. (***N*** = 5)	3–6 mo. (***N*** = 2)
Received formula recommendation or prescription from health provider					
Neither	10 (34.5)	4 (40.0)	3 (30.0)	2 (20.0)	1 (10.0)
Received recommendation without prescription	6 (20.7)	2 (33.3)	3 (50.0)	1 (16.7)	0
Received recommendation and prescription	13 (44.8)	6 (46.2)	4 (30.8)	2 (15.4)	1 (7.7)
Reasons reported for initiating formula use^a^					
Concerns about infant’s weight gain	12 (41.4)	3 (25.0)	4 (33.3)	4 (33.3)	1 (8.3)
Perceived breast milk insufficiency	8 (27.6)	3 (37.5)	4 (50.0)	1 (12.5)	0
Breastfeeding difficulties following Cesarean	4 (13.8)	4 (100.0)	0	0	0
Mother’s employment	2 (6.9)	0	1 (50.0)	0	1 (50.0)
Concerns about infant health following illness	2 (6.9)	2 (100.0)	0	0	0
Infant was LBW	1 (3.4)	0	1 (100.0)	0	0

^a^ Reported as the primary reason for formula use

^b^ Column percentages

^c^ Row percentages

At the same time, a number of other factors contributed to women’s decision-making around infant feeding, as displayed in Table 4. Twelve (41.4%) mothers expressed concerns about their infant’s weight gain as the primary reason for initiating infant formula use. Eight (27.6%) mothers mentioned the perception of insufficient breast milk as their main motive, describing such experiences with the phrases, “I didn’t have milk” (“*No tenía leche*”) and “[The baby] wasn’t filling up with my milk” (“*No se llenaba con mi pecho*”). As displayed in Table 4, nearly all of the participants who perceived breast milk insufficiency, initiated infant formula use before their infants reached 1 month of age; in contrast, more than 40% of women who reported concerns about their infant’s weight gain initiated infant formula use following the infant’s first month. Several mothers who had undergone a Cesarean delivery discussed the difficulties of starting to breastfeed after their infants received infant formula in the hospital and attributed their decisions to continue formula use to those experiences. Finally, two mothers reported initiating infant formula use after the infant experienced a bout of illness, and another two mothers stated that the need to return to work before the infant was 6 months old was the impetus for using formula.

Although Table 4 presents mothers’ primary motives in distinct categories, it should be noted that mothers’ decision to begin using infant formula generally arose from the interplay of several different factors. For example, Mercedes, a 21-year-old first-time mother, recalled that after giving birth at the main local hospital, a doctor told her, “*if she [the baby] does not fill up with my milk, that I should help her with formula*.” Upon returning home, Mercedes initially breastfed exclusively, yet during her baby’s third week of life, she grew concerned: “*I was feeding her and putting down to sleep, but after three minutes, five minutes, she would wake up*.*.. because she wasn’t filling up*.” At that point, Mercedes decided to begin mixed feeding.

### Initial infant formula brand selection

Most study participants who had received a health provider’s infant formula recommendation or prescription stated that they initially selected the particular brand that he or she had endorsed, regardless of its cost relative to other brands. For example, Antonia, a 41-year-old mother who lived in a one-room house in a recently constructed settlement without piped water or a bathroom, recalled being prescribed *S-26* *GOLD*, which was widely recognized as one of the most expensive formula brands. She explained, “*That has more... that one is more like breast milk, and the other formulas aren’t the same. That’s why the doctor prescribed me that one.”* When asked if she purchased this brand following that clinical encounter, Antonia responded “*I had to buy it, no matter what.”* Other study participants also recalled that doctors would emphasize the strengths of a particular infant formula brand over the alternatives while providing a formula recommendation or prescription. Raquel, a 28-year-old mother who began mixed feeding her infant at 3 weeks of age, recalled that her doctor recommended *Enfamil Premium*: “‘*This milk is good,’ he told me. ‘It won’t disrupt his stomach—it won’t give him diarrhea. It costs a bit more but it’s better,’ he told me. So I bought that one.*”

Several mothers of newborns who had been fed infant formula in the hospital following a Cesarean delivery reported purchasing the same brand of infant formula that the hospital had provided. Thirty-year-old Mónica, for example, recalled that she was discharged from the hospital three days after having an emergency Cesarean; before leaving, “*First I went to the neonatal unit to ask them what milk they had been giving, since I didn’t have much milk, and he said that I should help her with Enfamil—‘we’ll give you this name,’ he said*.” According to study participants, other reasons for selecting certain types of infant formula included recommendations from pharmacists and familiarity with a particular brand.

### Consequences of formula expenses

Mothers described that as their infants grew, the costs of BMS became a source of stress, especially amidst increasing financial demands for other aspects of childcare. Leyda, a 21-year-old single mother who had been mixed-feeding since her daughter’s second week of life, shared her concerns about the costs of *Enfamil Premium 1* for her daughter who was 2 months old at the time: “*She’s growing and drinking more and more milk... if right now [the tin] lasts me three days, how will it be later on?”*

Faced with these challenges, mothers developed a variety of financial coping strategies, such as demanding more support from the father’s family, filing an official complaint to receive such support, and borrowing money. Pilar, an 18-year-old first-time mother, shared,

Enfamil, that’s costs me a lot—sixty soles and every four days! I bought it on Saturday. .. no more than four days, the milk lasts no more than four days. .. Ay, I’ve had to borrow. The truth is that right now I owe a lady—a neighbor—because I had to buy more [formula] and I didn’t know what to do*.*

Other participants mentioned changing their own eating habits to contend with the costs of formula. Carolina, a 28-year-old mother who began feeding formula to her twins during their first week of life, explained her decision to continue purchasing the same brand of formula: “*If [the doctor] is recommending that it’s good, and that it will be good for the baby, then what can I do? I had to work double—what can I do?... Where before I was eating two breads, I just eat one bread now.”*

### Decisions to change products

Commonly, mothers reported switching to a less expensive type of infant formula as the costs grew more prohibitive. This was the case for Veronica, a 37-year-old mother of five who had received free donations of *S-26* *GOLD* from a nurse at the government-sponsored hospital when her baby was a few days old. As she explained, “*And from there, when it ran out, everything my husband earned was going to buy, buy, and buy, and it was too much—the costs and everything, the diapers. There wasn’t enough money anymore.”* When her child was 3 months old, she switched to *Enfamil con Hierro*, the least expensive option from the *Enfamil* line. Yet within a week she reported her baby was badly constipated. To avoid wasting the newly purchased formula, she and her husband diluted it: “*Maybe you’re giving it to her too thick. Add a little more water,’ [my husband] told me. So I added a little more water—but just the same, she was constipated.”*

Several mothers recalled that they decided to change to follow-on formulas in light of financial constraints, since these were more affordable than infant formula. Participants described how these choices were largely influenced by input from their family members and friends, as well as observable outcomes in other children. For example, María, a 23-year-old mother of four, began purchasing *Enfagrow Premium*, which is meant for children > 12 months of age, when her child was approximately 1 month old. According to María, *Enfagrow Premium* was close to half the price of *Enfamil Premium 1*. She recalled feeling confident in this choice based on her sister’s experiences: “*My niece also used that one. She used that formula from when she was little until she was four years old. That’s why she’s bigger than my daughter [of the same age]*.” Other mothers of infants under 6 months of age began purchasing small cans of the follow-on formula *NAN 2,* due to its affordability and availability in reduced volumes.

In addition, several mothers recalled switching to commercial milk, the most economical option on the market, for their young infants. Participants described how such changes were often motivated by suggestions from their family members or social networks. For example, after feeding her son *Enfamil Premium* for more than 2 months, Raquel recalled that her mother recommended *Anchor* milk, based on her own childrearing practices:

My mamá says that she brought us up with Anchor when we were little. She says that I drank Anchor, that’s why she tells me, “Anchor is good.” I had a nephew that grew up well with Anchor and he still ended up as tall [as the other kids his age]—he was drinking pure Anchor. He didn’t breastfeed—just pure Anchor. .. That’s why she tells me, “Give him Anchor, and he’ll be like Juan—tall!”

*Anchor*, which is sold as a dehydrated powder and packaged in a cylindrical tin that resembles those of infant formula brands, was more commonly substituted for infant formula than other brands of milk sold as liquids. *Anchor* was even perceived to be infant formula in some cases, as one mother explained, “*We buy the formula that I have right now—Anchor—because it’s the cheapest one that there is.”*

### Judging the adequacy of purchased products

Upon switching to a less expensive type of BMS or to commercial milk, mothers recalled monitoring their infants’ reactions to ensure the acceptability of the new product. Rocío, a 33-year-old mother of four, had begun mixed-feeding her daughter with *NAN 1* when she was 2 weeks old, yet switched to *Anchor* when she was approximately 3 months old. She described her reservations about making this change, knowing that health providers did not approve of it.

I was scared, as the saying goes, but I made myself strong. I made myself really strong; I have to give her this one because it’s cheaper. I felt calm—one day went by, then two days, then three days, and then I said “Ok, my daughter isn’t going to get sick, everything is alright then.” Because if it hits you, it hits you on the first and second days, but not on the third and fourth days. She had already adapted*.*

Others perceived that certain recommendations did not apply specifically to their infant. As Victoria, a 32-year-old mother of two who began feeding her son *Gloria* milk when he was about 1 month old, commented, *“[The nurses] always say that it’s bad, they’re always telling you that it’s bad – ‘How are you going to give him this?’ But they say that not all babies are the same... I give him Gloria milk and it’s fine. I thank god, with Gloria milk he’s doing well.”*

**Fig. 1:**
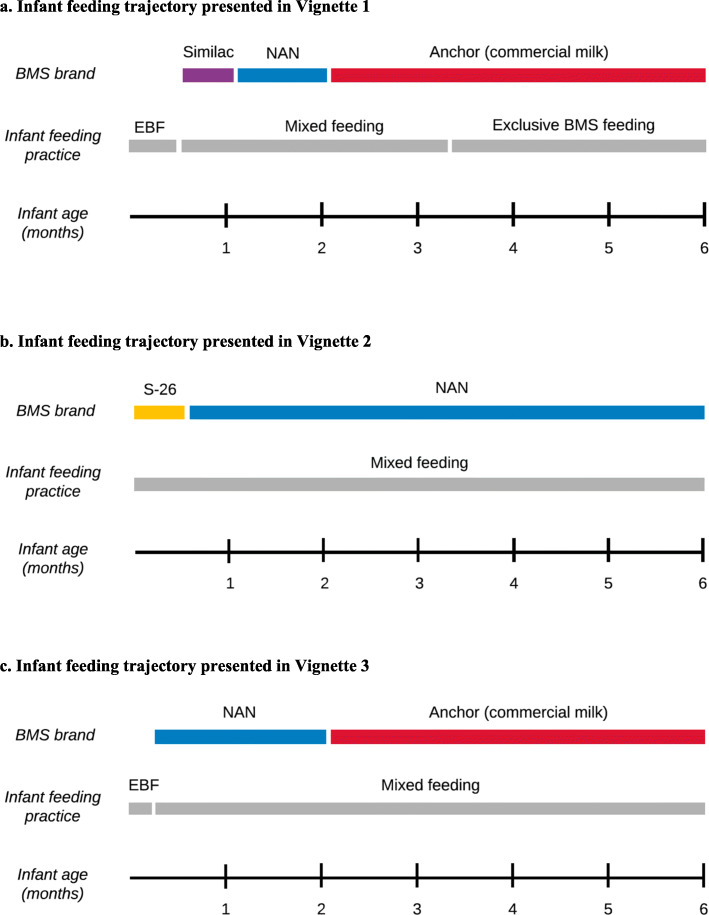
Infant feeding trajectories presented in Vignettes 1-3

The following vignettes, grounded in the interviews conducted with each participant, illustrate the interplay of the themes discussed above. The trajectories of these three mothers’ feeding and BMS purchasing behaviors are summarized in Figs. 1a-c.

### Vignette 1: Rosario, 14 years old, with a four-month-old infant

Rosario and her baby lived in a small two-bedroom home with six other family members, including her two-year-old twin sisters. After giving birth in the large government-funded hospital, 14-year-old Rosario struggled to breastfeed her newborn, and a doctor suggested that she purchase *Similac* formula if the difficulties were to continue. She recalled, *“He told me, ‘If you don’t have milk, or if your baby doesn’t latch on to your breast,’ he told me, ‘you have to give her formula because she’s going to lose too much weight, and it will be difficult to recover it,’ he told me.”*

Rosario breastfed exclusively for 2 weeks, but when she grew worried that her baby was not gaining weight quickly enough, she asked the baby’s paternal grandfather to buy a tin of *Similac* and a bottle. The next time that she was responsible for purchasing the formula, she purchased a different brand, *NAN 1*, because it was more affordable, and her baby responded well. “*He was doing well with NAN, he was gaining weight—his weight was really good.”* Yet she went on to say, *“But then I didn’t have enough money. With the packages of diapers, the baby wipes... and I only had 30 soles left.”*

Due to the growing costs of raising her baby, she soon realized that feeding her *NAN* *1* was not viable. To supplement the funds provided by the baby’s father, who was also under-age, Rosario was relying on help from her mother and her mother’s partner. At this point, the baby’s aunt suggested that she purchase *Anchor*, since it was almost half the price as *NAN* *1*: “*She told me, ‘Why don’t you feed her Anchor, which is 27 [Nuevos Soles]? I’m feeding my son Anchor,’ she told me, ‘and look, he has gained a good weight*.”

When Rosario was able to purchase a large tin of *Anchor* along with the diapers and baby-wipes that she needed for the week, her worries subsided. She felt further relieved when her daughter did not exhibit any signs of illness upon starting to consume *Anchor*: “*She took it well. She didn’t get sick, her stomach wasn’t constipated—she was fine. And she’s better than fine--rightly so, she’s gotten even bigger cheeks!”* By 4 months of age, the child was consuming *Anchor* exclusively, given that Rosario’s breast milk supply had diminished.

### Vignette 2: Blanca, 40 years old, with a two-month-old infant

Blanca was an illiterate mother of three who grew up in an Andean farming community and moved with her family to *Villa El Salvador* as an adolescent. Due to problems during delivery, her newborn stayed in the hospital for most of his first month of life, where the doctor ordered Blanca to purchase several tins of *S-26* *GOLD* for in-hospital feeding. When she began breastfeeding, she was surprised at the difficulty of providing enough breast milk, since she had never faced this problem with her older children. She recalled that the doctor said, “*Since you’re older—since you’re 40 years old—maybe that’s why you don’t have much milk*,” and suggested that she continue feeding her son *S-26* *GOLD* following hospital discharge.

Upon bringing him home, she was concerned about the continued costs of infant formula, explaining, “*I didn’t have money... and money is what makes the rules.”* Her financial worries were heightened by the debts that she now had with friends and neighbors who had lent money to cover the unexpected costs of her emergency Cesarean delivery. When she went to the pharmacy to ask about different infant formula brands, she learned that the small container of *NAN* cost 20 soles less than *S-26* *GOLD*. She recalled, “*That’s why I said—as I don’t know how to read—I said, ‘this one will be the same.*” However, the pharmacy clerk advised against switching brands: “*He says to me, ‘Ma’am, what the doctor is recommending, that’s what you have to give him, because he was born drinking this milk.*” Yet given her household’s economic circumstances, this was not an option for her.

After Blanca started feeding her infant with *NAN*, he began to have diarrhea, causing concern. At the time of the interview, he had been ill for several days; Blanca explained sorrowfully,

I want him not to have diarrhea. He has diarrhea and sometimes I worry, I say, ‘Instead of gaining weight, he’s going to lose it,’ and I don’t want that. That’s what I don’t want. I want him to have a good weight, to not get sick anymore*.*

When asked if she was planning to continue feeding her child *NAN*, she was resigned to the impracticality of doing anything else: *“I have to finish it—what other option do I have? I’m not going to put everything to waste for nothing. Until I can buy that one [S-26 GOLD], what else can I do?”*

### Vignette 3: Sonia, 34 years old, with a seven-month-old infant

Sonia grew up in the central highlands of Peru and moved to a young settlement in *Villa El Salvador* eleven years ago in search of work. After giving birth to her third daughter in the local maternal-child health center, 34-year-old Sonia and her new baby stayed there for two days because, as she recalled, she was at risk of anemia. Sonia began breastfeeding her daughter right away, stating that “*as soon as you leave the room, they send you to lay down in the bed and you have to start—start breastfeeding the baby... they force you to*.” Yet after returning home, she grew worried that she was not producing enough breast milk, noting that her baby continued to cry even after feeding.

When her child was five days old, she decided to purchase a tin of *NAN* formula, given that it was the most recognizable brand. During a postnatal visit, when Sonia told a nurse that she had begun mixed feeding, the nurse grew visibly annoyed. Sonia described their conversation: “*I say, ‘but if it’s milk, miss, it’s the same.’ ‘No,’ she says, ‘breast milk is much better than formula milk.’ So I say, ‘Miss, but what can I do if I don’t have enough breast milk? I can’t do anything!’*

Before long, Sonia grew aware of the mounting costs of formula, as one tin of *NAN* lasted only three days. In addition, she had to replace her thermos, used for transporting prepared formula, on two occasions after it broke. Sonia’s mother, who lived in the same home, chastised her: ‘*She says to me now, ‘You’re not going to have another baby, because with one more, in the end you won’t have anything left!*’ Sonia continued to work once or twice per week, leaving the baby with her 16-year-old daughter. As she explained,

When you work, at least a little something comes in, even if it is just pennies. If I’m sitting at home, nobody—there’s no one giving you [money]. Sometimes your husband gives you something, but it’s not enough for anything.

Nevertheless, it proved difficult to afford *NAN*. When the baby was about 2 months old, Sonia bought a container of *Anchor* milk, which cost far less than *NAN* and lasted 15 days. Although her mother supported this decision, several friends and neighbors warned her against it. Nevertheless, Sonia recalled, “*But I told them that for the sake of my pocketbook, I’m going to keep giving her Anchor*. *And I have—to this day*.”

## Discussion

**Fig. 2:**
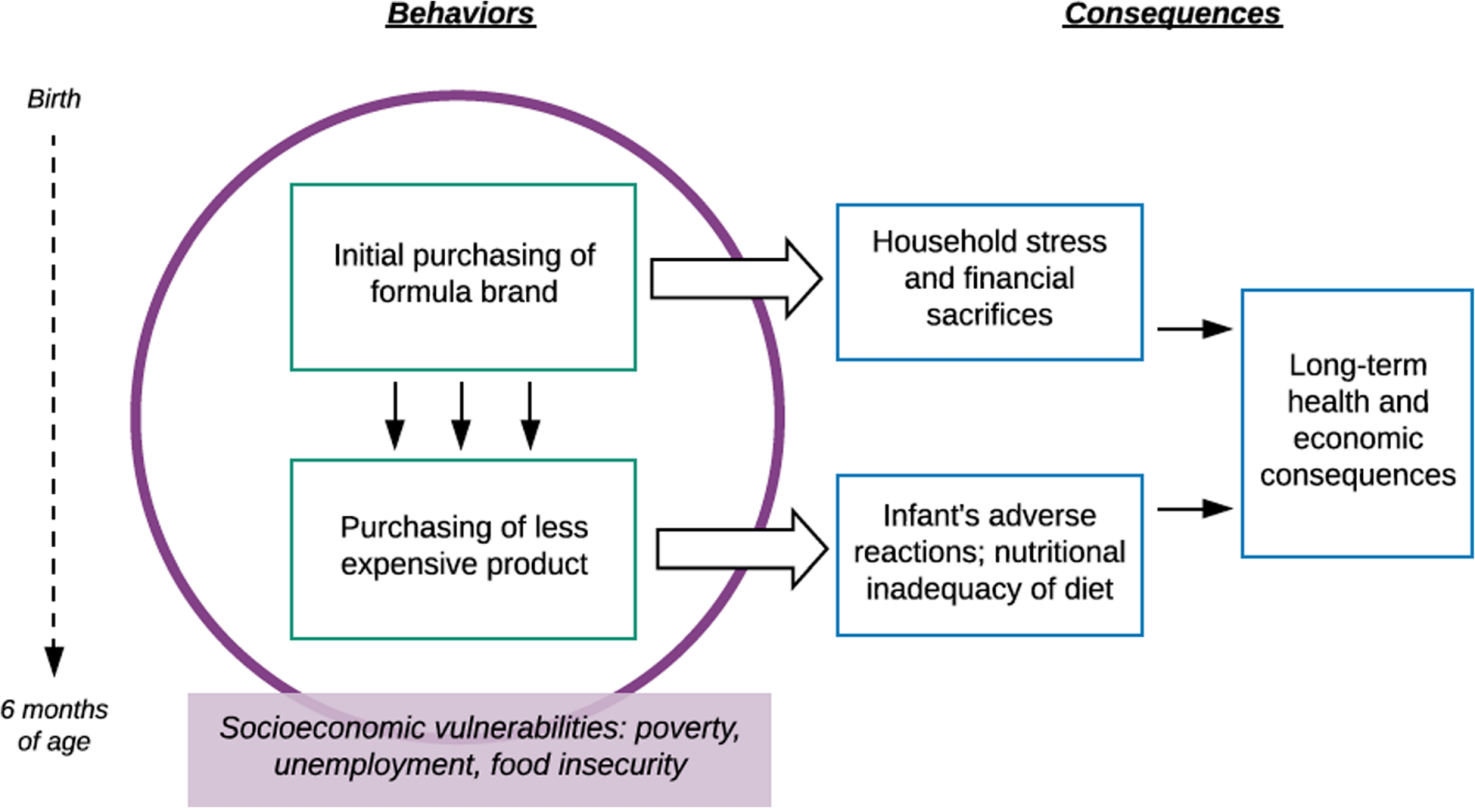
Descriptive model of findings

Through the voices of vulnerable mothers, we have aimed to deepen readers’ understanding of how women make choices surrounding infant feeding during the period of time when EBF is recommended. The descriptive model presented in Fig. 2 summarizes our findings related to BMS purchasing behaviors and their repercussions. We demonstrated that the incorporation of BMS into a family’s weekly expenses may significantly disrupt the household economy, especially given that the amount of BMS required for an infant increases with time. For many mothers, this pressure was further heightened by the perceived need to adhere to a health provider’s recommendation or prescription for a specific brand of infant formula, which was often markedly more expensive than the alternatives. Thus, many women found themselves taking measures that could threaten their well-being in order to purchase top-of-the-line formula, such as borrowing money or consuming less food themselves. In the context of economic anxieties, participants also revealed the tendency to switch to less expensive products, including follow-on formula and commercial milk, over time.

The decisions to begin purchasing less expensive products may generate both short- and long-term health risks, as depictured in Fig. 2. The immediate aftermath of changing an infant’s food source often involves gastrointestinal symptoms such as constipation or diarrhea, which can interfere with nutrient absorption. These issues were experienced by the children of Veronica and Blanca (Vignette 2); in both cases, the mothers continued feeding their infants the newly purchased products to avoid wasting money. In addition, the feeding of follow-on formula to infants under 6 months of age is of concern. According to the WHO, follow-on formula is “unsuitable” as a breast milk replacement for infants 0–6 months of age given that “current formulations lead to higher protein intake and lower intake of essential fatty acids, iron, zinc and B vitamins” than what is recommended for adequate infant growth and development [41].

Even more pronounced are the risks associated with feeding commercial milk such as *Anchor* to young infants, as Rosario (Vignette 1) did. Consumption of commercial milk increases the likelihood of developing iron deficiency anemia due to its low iron content and the risk of occult intestinal tract blood loss [51, 52]. In addition, the high concentrations of protein and minerals such as sodium, potassium, and phosphate may lead to higher renal solute load, placing infants at risk of dehydration [53]. Yet these health threats are unobservable in the short term. As several study participants demonstrated, it was widely recognized that commercial milk was not recommended for infants younger than 6 months, yet mothers often judged it as safe in the absence of an immediate adverse reaction in their infant.

### Implications for breastfeeding promotion

In peri-urban Lima, our participants’ experiences suggest that there is a need for the health system to devote more resources towards educating and supporting mothers to breastfeed. First, greater emphasis must be placed on the negative health sequelae and financial burden associated with breast milk substitutes. Our study participants revealed that they often used immediate, observable events, such as an infant’s weight gain or the lack of diarrhea, to ease their apprehensions surrounding certain types of breast milk substitutes. These experiences suggest that the long-term health risks of BMS do not factor heavily into women’s attitudes towards infant feeding options. Researchers and breastfeeding advocates have pointed to the public health field’s messaging surrounding EBF promotion, where “breast is best” has become the dominant slogan, to explain this trend [54, 55]. Specifically, they argue that framing breastfeeding as the “best” choice implies that feeding with BMS is normal and acceptable [56]. To address this issue, health providers should invoke “loss framing” to a greater extent by counseling women and other family members that may accompany them during delivery and postnatal visits in the risks of BMS use for both child health and for the household’s economic well-being [44, 55].

Second, in addition to education, breastfeeding promotion efforts should provide women with tools to decline BMS when they are offered or encouraged to use it. Data from our participants as well as several other studies have demonstrated that women’s family members and social networks, particularly the infant’s grandmother, can be powerful influences on feeding practices, often pressuring mothers to make the same decisions that their generation did previously [57–59]. In these contexts, women would benefit from having high BMS refusal self-efficacy. Refusal self-efficacy, defined as a person’s belief in their ability to resist something offered to them, has been demonstrated to predict and protect against other unhealthy behaviors including tobacco smoking and alcohol consumption [60–62]. Health providers should endeavor to strengthen women’s BMS refusal self-efficacy by helping them practice the exact words and reasoning for declining BMS. These resilience skills should be geared towards refusing BMS offers not only from household members, but also from doctors or infant formula company representatives within health facilities themselves, given that marketing regulations in Peru and elsewhere have not succeeded in keeping infant formula representatives out of the health sector [18, 63]. Building refusal self-efficacy may be particularly relevant during individual or group antenatal care counseling in order to ensure that women have these skills before they become necessary.

Third, our study findings indicate missed opportunities for women to return to EBF after a period of mixed feeding, or for relactation after breastfeeding has stopped. Our participants’ experiences suggest that they perceived the decision to feed an infant BMS as irreversible. When the financial burden became untenable, these women did not discuss or attempt to return to EBF; rather, switching to a less expensive BMS brand or commercial milk product appeared as the only option. However, studies conducted in other sites have shown that it is common for mothers to return to EBF after feeding their children other liquids or foods for one or more days during the first 6 months of a child’s life [64, 65]. In the Peruvian Amazon, Ambikapathi and colleagues (2016) analyzed the daily feeding practices of a cohort of 268 mother-infant dyads and demonstrated that infants under 6 months experienced an average of three separate episodes of EBF; thus, mothers’ capacity to return to EBF after cessation resulted in important gains in days of exclusive breastfeeding [65]. In peri-urban Lima, there is a need for health providers to support and counsel women to breastfeed even after BMS use has begun. Extensive evidence indicates that dedicated counseling, along with strong motivation from mothers, facilitates relactation and the return to exclusive breastfeeding [66–68]. Providers should explain to mothers that BMS use can be temporary and short-term, if needed, and they should provide appropriate counseling to support EBF after it has been interrupted by explaining how a woman’s breast milk supply may be built up again after decreasing. For mothers who have begun feeding BMS to their infants, it is possible that emerging financial concerns could catalyze efforts to return to EBF or to relactate.

### Limitations

This study was limited by several factors. Participants were interviewed on only one or two occasions rather than longitudinally. Although open-ended questions were employed to encourage storytelling, the timing of the interviews may have introduced an element of recall bias. In addition, a common critique of qualitative research is that the researcher’s own experiences may influence the interpretation of the findings [69]. However, several measures were taken to ensure analytic rigor and the credibility of our analysis, such as eliciting feedback from field workers and respondent validation. Finally, the results of this study may not be generalizable outside of the specific setting in peri-urban Lima. Yet our study area may be comparable to peri-urban communities in Peru and other Latin American countries, especially with regards to the socio-economic constraints that typify shantytown developments, increasing the transferability of our findings [48]. This is important given the rapid growth of urban and peri-urban areas throughout LMICs, and the fact that the infant formula industry is increasingly targeting these markets [15, 20, 70].

## Conclusions

This qualitative study reveals that the threats associated with mixed feeding during early infancy include significant disruption to the household economy, and the introduction of new health risks among children who are fed follow-on formula or commercial milk. The breastfeeding education and support that mothers receive during their clinical encounters depend largely on the quality of health providers’ training. Providers must be educated on the full set of risks associated with recommending or prescribing BMS when not medically indicated, including the possibility that mothers may switch to follow-on formula or commercial milk for young infants due to financial constraints, and be equipped to effectively counsel women on breastfeeding technique, positioning, and problem-solving.

Future research should explore the extent to which medical and nursing curricula in Peru provide training in all aspects of breastfeeding support, including BMS refusal self-efficacy, maintenance of breastfeeding, and relactation. Additional research at health facilities, including direct observations during antenatal, delivery, postnatal, and well-baby care, will facilitate a better understanding of how providers’ training curricula could be improved and expanded. Ensuring health providers’ competencies in these aspects of counseling is essential to curbing trends towards infant formula feeding in Peru and other low-resource settings.

## Data Availability

The transcripts analyzed during the current study can be made available by the corresponding author on reasonable request.

## Abbreviations

BMS: Breast milk substitute
EBF: Exclusive breastfeeding
HFIAS: Household Food Insecurity Access Scale
IDI: In-depth interview
LMIC: Low- and middle-income country
WHO: World Health Organization
